# Supplementary material for: In situ 10-cell RNA sequencing in tissue and tumor biopsy samples
Source: Sci Rep. 2019 Mar 20;9:4836. doi: 10.1038/s41598-019-41235-9 (PMC6426952; doi:10.1038/s41598-019-41235-9)
Supplement: Supplementary file 1 — Supplementary Figures and Tables [file 41598_2019_41235_MOESM1_ESM.pdf]

SUPPLEMENTARY DATA

In situ 10-cell RNA sequencing in tissue and tumor biopsy samples

Shambhavi Singh[1†], Lixin Wang[1†], Dylan L. Schaff[1], Matthew D. Sutcliffe[1], Alex F. Koeppel[2], Jungeun Kim[3], Suna Onengut-Gumuscu[4], Kwon-Sik Park[3], Hui Zong[3], Kevin A. Janes[1,4,5*]

[1] Department of Biomedical Engineering
[2] Bioinformatics Core
[3] Department of Microbiology, Immunology & Cancer Biology
[4] Center for Public Health Genomics
[5] Department of Biochemistry & Molecular Genetics, University of Virginia, Charlottesville, VA. 22908

* To whom correspondence should be addressed. Tel: +1 434 243 2126; Fax: +1 434 982 3870; Email: kjanes@virginia.edu

† The authors wish it to be known that, in their opinion, the first two authors should be regarded as joint First Authors

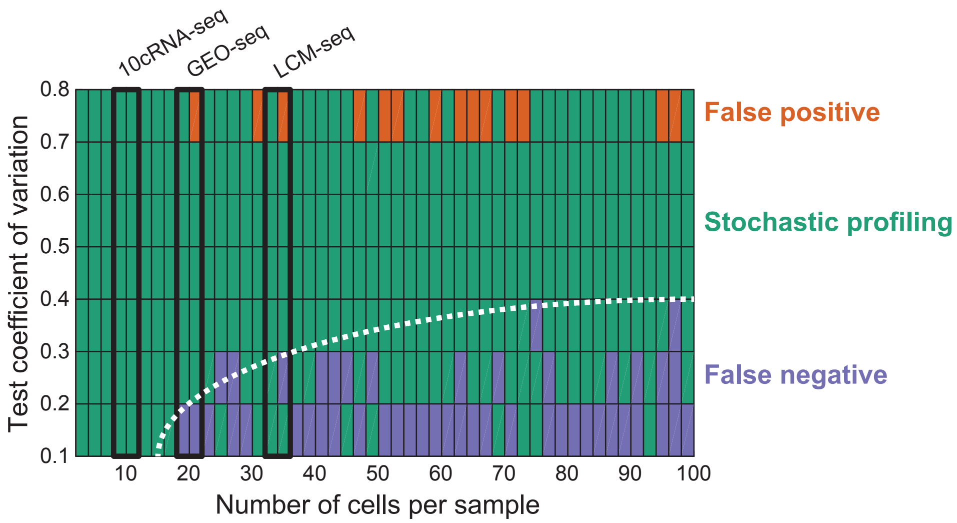

Supplementary Figure S1. Population averaging obscures single-cell regulatory heterogeneities in pools of more than ~15 cells. Monte Carlo simulations[1] of stochastic-profiling experiments are shown for 25 random samples, an expression fraction of 50%, a reference coefficient of variation of 0.3, and a fold difference in regulatory states of 5. False positives (orange) arise when a one-state gene with high variance relative to the reference distribution is incorrectly scored as having two regulatory states. False negatives (blue) arise when a two-state gene with low variance relative to the reference distribution is incorrectly as scored as having one regulatory state. Effective stochastic profiling (green) occurs when two-state genes are correctly scored as heterogeneously regulated. Cell input requirements for 10cRNA-seq are shown compared to applications of GEO-seq[2] and LCM-seq[3]. Note the increases in false negatives for larger test variances observed with larger number of cells per sample.

hGFAP-Cre MADM brain

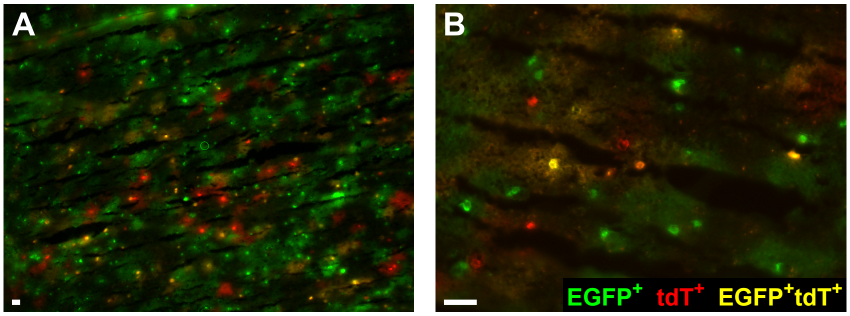

Supplementary Figure S2. Fresh cryoembedding and 70-95-100% ethanol dehydration retains sufficient EGFP fluorescence and localization to identify single cells alongside tdT. Tissue preparation was performed with a mosaic analysis of double markers (MADM) animal expressing Cre under control of the hGFAP promoter to label multiple brain lineages with EGFP (green), tdT (red), or both (yellow). **(A)** Low- and **(B)** high-magnification images were captured with the factory-installed color camera on the Arcturus XT LCM instrument. Red and green spectral channels were separated, false colored, and merged to generate final images. Scale bar is 25 µm.

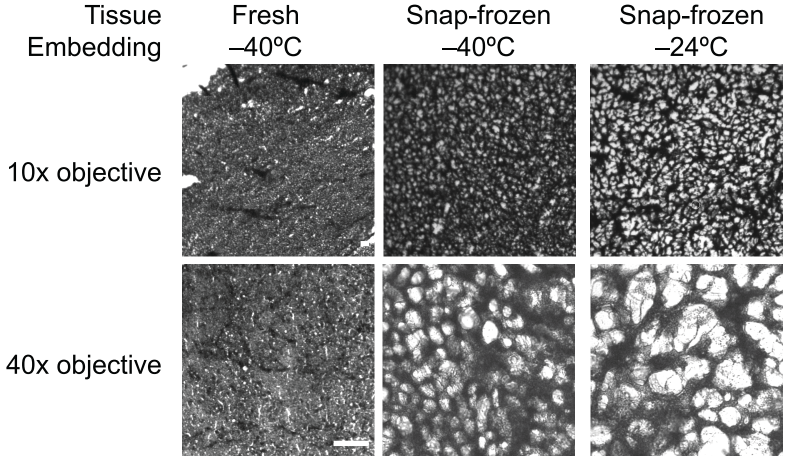

Supplementary Figure S3. Fresh cryoembedding preserves tissue integrity better than snap-frozen alternatives. Brain samples from *Cspg4-CreER;Trp53*$^{F/F}$*;Nf1*$^{F/F}$*;Rosa26-LSL-tdT* animals were **(A)** freshly cryoembedded in Neg-50 medium with dry ice-isopentane (–40ºC), **(B)** snap-frozen in dry ice-isopentane and then cryoembedded, or **(C)** snap-frozen and slowly cryoembedded in a cryostat (–24ºC). Low- and high-magnification images were captured with the factory-installed color camera on the Arcturus XT LCM instrument and converted to grayscale. Scale bar is 25 µm. Images of tdT fluorescence from the same sections are shown in Fig. 2.

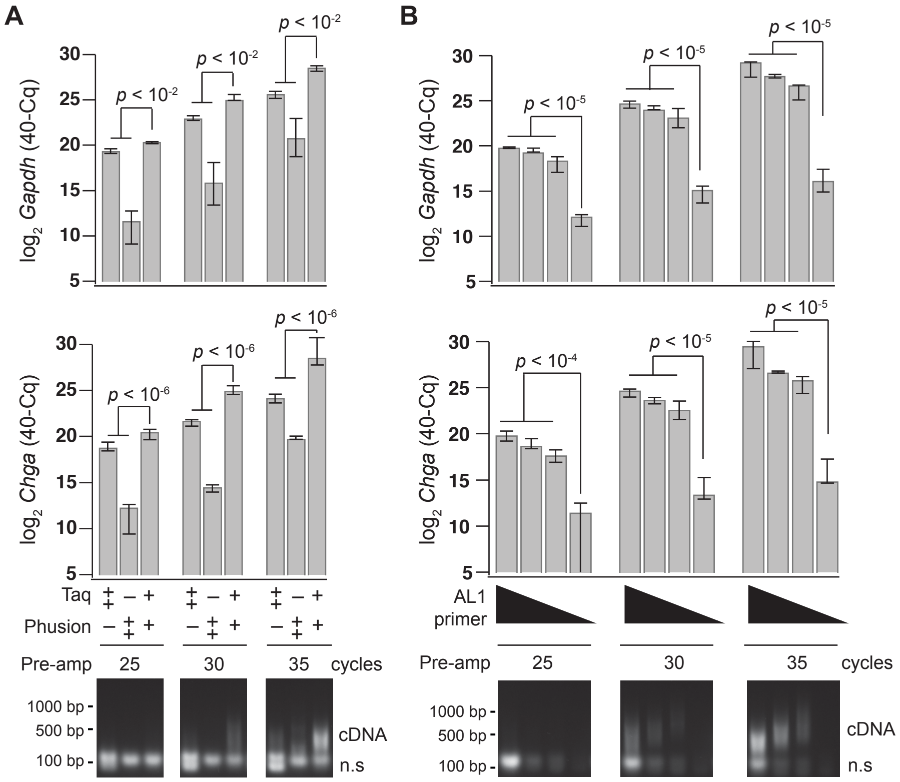

Supplementary Figure S4. Improvements with the Taq–Phusion polymerases blend generalize to murine small-cell lung cancer cells. Cells were obtained by LCM and split into 10-cell equivalent amplification replicates. **(A)** Poly(A) PCR was performed with 25 µg of AL1 primer with Taq alone (10 units), Phusion alone (4 units) or Taq/Phusion combination (3.75 units/1.5 units). **(B)** Poly(A) PCR was performed with either 25, 5, 2.5 or 0.5 µg of AL1 primer and the Taq–Phusion blend from (A). Above—Relative abundance for the indicated genes and preamplification conditions was measured by quantitative PCR. Data are shown as the median inverse quantification cycle (40–Cq) ± range from n = 3 amplification replicates and were analyzed by two-way (A) or one-way (B) ANOVA with replication. Below—Preamplifications were analyzed by agarose gel electrophoresis to separate poly(A)-amplified cDNA from nonspecific, low molecular-weight concatemer (n.s.). Lanes were cropped by poly(A) PCR cycles for display but were electrophoresed on the same agarose gel and processed identically. The uncropped image is shown in Supplementary Fig. S13B.

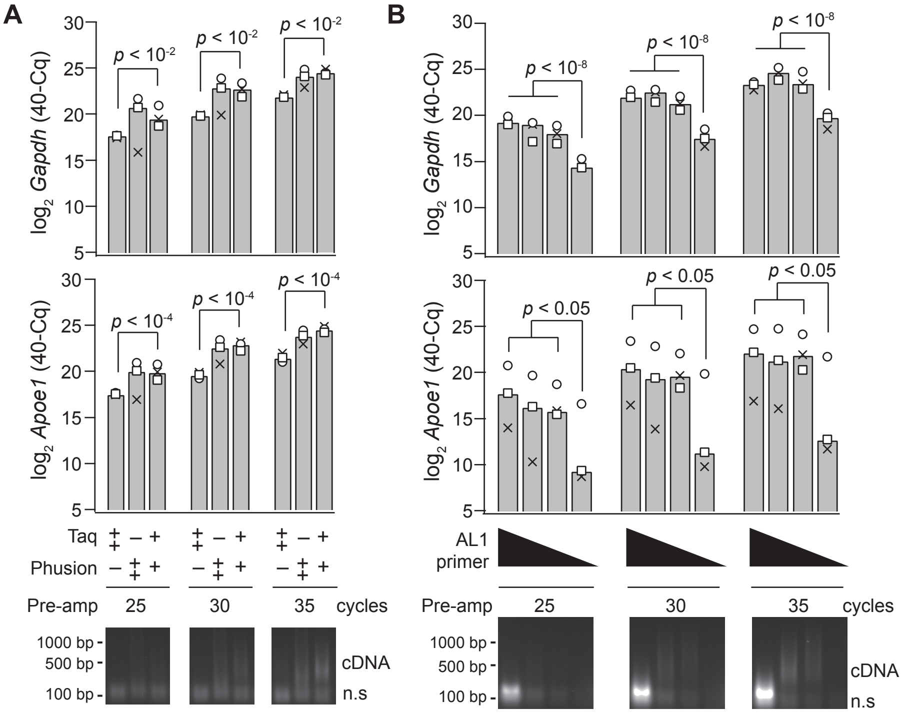

Supplementary Figure S5. Improvements with the Taq–Phusion polymerases blend generalize to murine tdT-labeled oligodendrocyte precursor cells. Cells were obtained by fluorescence-guided LCM and split into 10-cell equivalent amplification replicates. **(A)** Poly(A) PCR was performed with 25 µg of AL1 primer with Taq alone (10 units), Phusion alone (4 units) or Taq/Phusion combination (3.75 units/1.5 units). **(B)** Poly(A) PCR was performed with either 25, 5, 2.5 or 1 µg of AL1 primer and the Taq–Phusion blend from (A). Above—Relative abundance for the indicated genes and preamplification conditions was measured by quantitative PCR. Data are shown as the median inverse quantification cycle (40–Cq) ± range from n = 3 replicates collected over three separate LCM acquisitions (markers) and were analyzed by two-way ANOVA with replication. Below—Preamplifications were analyzed by agarose gel electrophoresis to separate poly(A)-amplified cDNA from nonspecific, low molecular-weight concatemer (n.s.). Lanes were cropped by poly(A) PCR cycles for display but were electrophoresed on the same agarose gel and processed identically. The uncropped image is shown in Supplementary Fig. S13C.

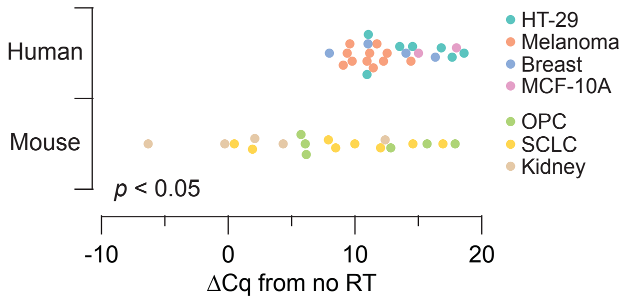

Supplementary Figure S6. Prevalence of genomic DNA contamination during poly(A) amplification of mouse tissue. Differences in quantification cycles between LCM samples ± reverse transcription (ΔCq from no RT) are shown for various genes in HT-29 cells (human colon adenocarcinoma), primary human melanoma and breast cancer, and MCF-10A cells (human breast epithelial) compared to mouse oligodendrocyte precursor cells (OPC), mouse small-cell lung cancer cells (SCLC), and mouse kidney cells isolated by LCM. Human-mouse differences were assessed by rank-sum test.

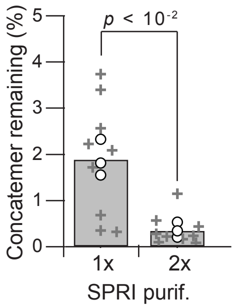

Supplementary Figure S7. Two rounds of SPRI bead purification reduce low molecular-weight contaminants from 10-cell reamplifications of mouse small-cell lung cancer cells. Data are shown as the mean (gray) of n = 3 independent reamplifications (circles) each purified three times (+). Differences were assessed by two-way ANOVA with replication. The uncropped gel image used for concatemer densitometry is shown in Supplementary Fig. S13F (lower).

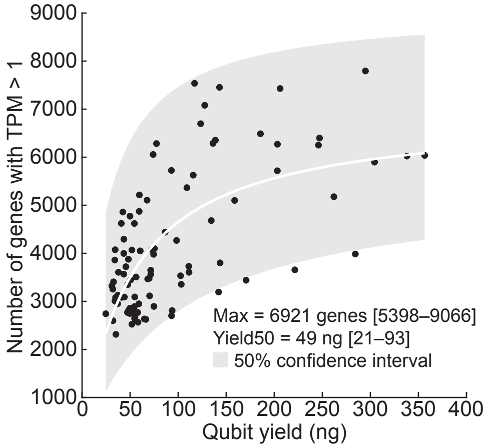

Supplementary Figure S8. Maximal gene-detection sensitivity requires an SPRI bead yield of at-least 200 ng poly(A) cDNA. Low-coverage RNA sequencing of mouse oligodendrocyte precursor cells or transformed derivatives (*n* = 96) was used to relate gene-detection sensitivity to SPRI bead yield quantified by Qubit fluorescence through a hyperbolic function with the indicated parameters (Max, Yield50).

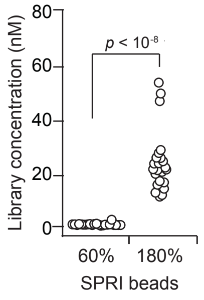

Supplementary Figure S9. Higher SPRI bead ratio is essential for purification of tagmented libraries. TapeStation concentrations of sequencing libraries following tagmentation and purification with either 60% or 180% [vol/vol] beads. Differences in library concentrations were assessed by paired two-tailed *t* test.

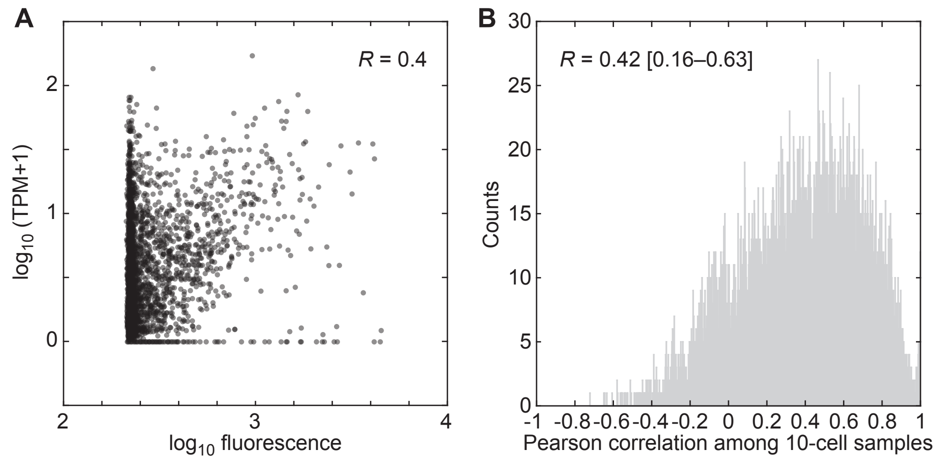

Supplementary Figure S10. Significant technical and biological covariation between BeadChip microarray and 10cRNA-seq. **(A)** Variably detected genes remain correlated between transcriptomic platforms. Genes with at-least one pool-and-split TPM = 0 (n = 3256 genes) were plotted versus BeadChip fluorescence. **(B)** Significant sample-to-sample correlations between independent 10-cell pools (n = 18) measured by BeadChip microarray and RNA-seq. The median log-scaled Pearson correlation (R) is shown with the interquartile range in brackets for 7713 genes.

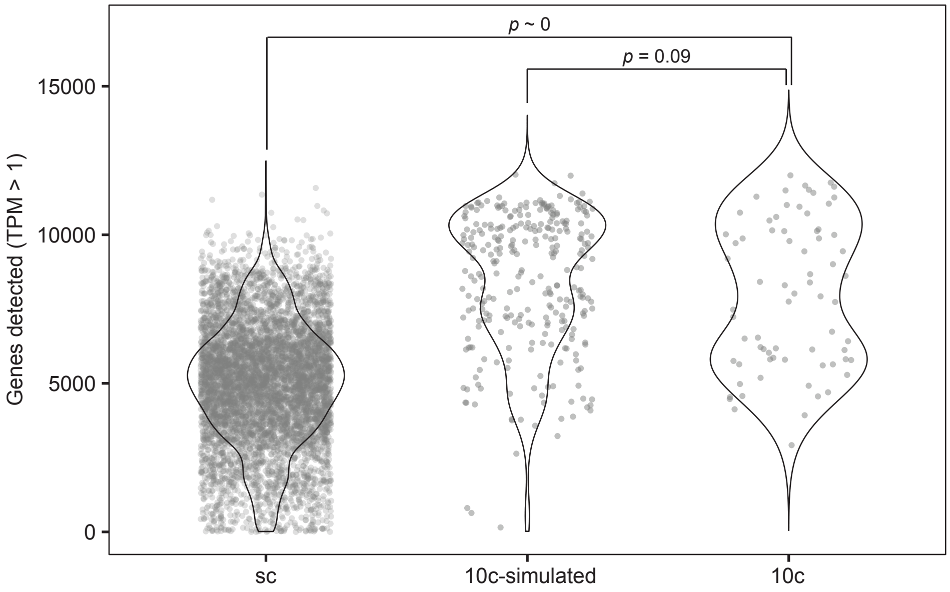

Supplementary Figure S11. Gene-detection sensitivity increases predictably to that of 10cRNA-seq when scRNA-seq data are aggregated as 10-cell pools. Single cells from various scRNA-seq datasets (described in Fig. 8) were randomly sampled and grouped with their nine nearest neighbours, and dropouts were modelled using a binomial process for RNA-to-cDNA conversion (n = 48 random samples for each of six datasets; see Methods). Gene detection from the original scRNA-seq datasets (sc, reprinted from Fig. 8), the simulated 10-cell pools (10c-simulated), and 10cRNA-seq (10c, reprinted from Fig. 8) were compared by permutation test.

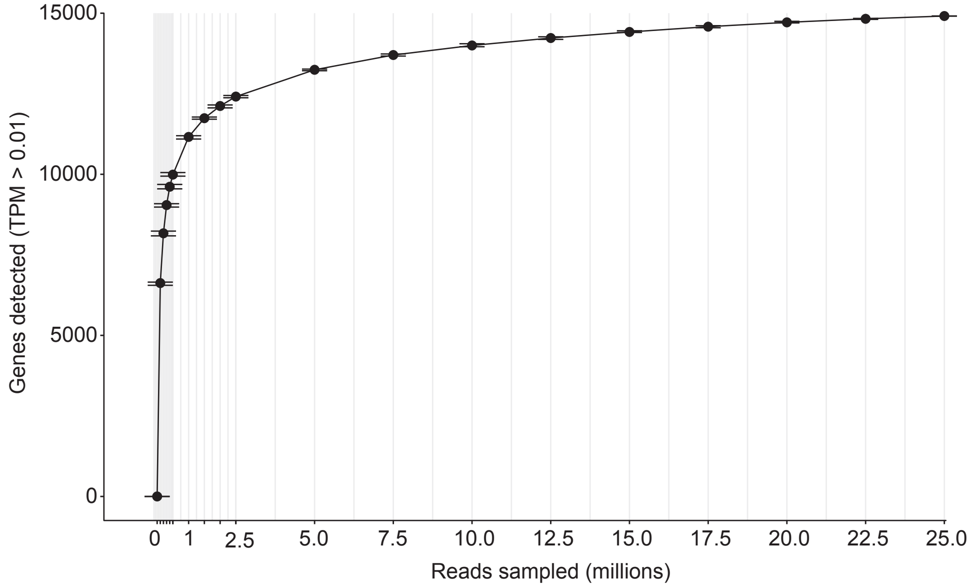

Supplementary Figure S12. 10cRNA-seq gene detection saturates above 5 million reads per sample. 10cRNA-seq reads from MCF10A-5E cells were aggregated, randomly sampled at the indicated depth, and aligned. Data are shown as the median number genes detected (TPM > 0.01) ± range of *n* = 10 random draws per depth.

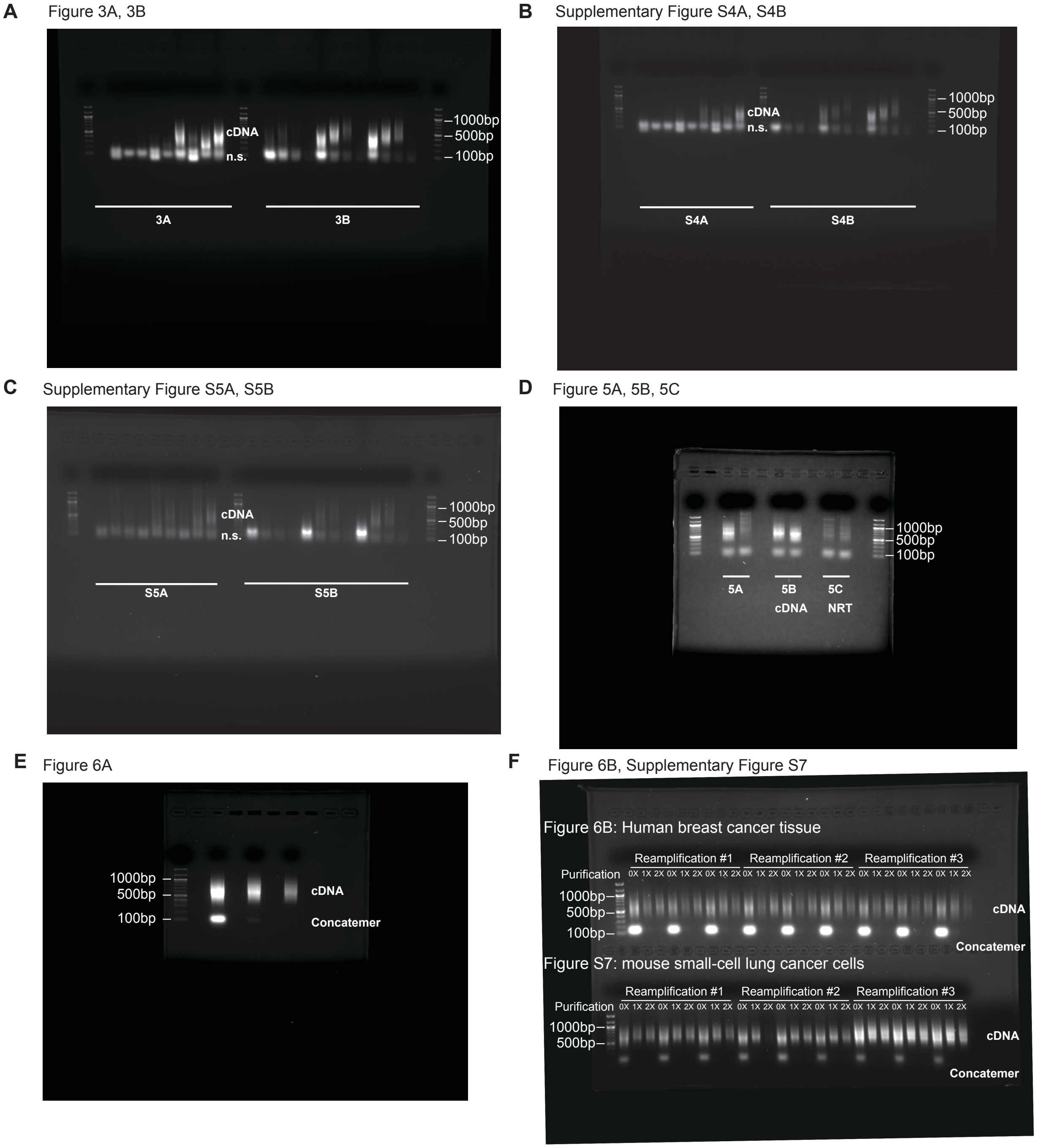

Supplementary Figure S13. Uncropped images for agarose gels with molecular weight markers. **(A–C)** Bands were cropped by poly(A) PCR cycles as indicated in the main figures. **(D)** The gel was first cropped lengthwise to generate electrophoretic traces and then cropped by condition. **(E)** All bands were presented in Fig. 6. **(F)** The gel used for densitometry of bands corresponding to concatemers in Fig. 6B (top) and Supplementary Fig. S7 (bottom). Sets of three samples are in the following order: unpurified, 1x purified, 2x purified. Each set of three conditions was repeated three times for three independent re-amplifications, resulting in nine quantified samples per purification condition.

Supplementary Table S1. Published scRNA-seq datasets used in this study.

Study	Tissue	Method	Read length	Read depth (10^6)*
GSE75330	Mouse OPC	Full length	50-bp single end	1.34 [0.6–8.4]
GSE60361	Mouse OPC	Full length	50-bp single end	1.85 [0.6–8.4]
GSE103354 Plate-based	Mouse lung	Full length	75-bp paired end	0.32 [0.06–0.6]
GSE103354 Droplet	Mouse lung	3'-end	75-bp single end	0.07 [0.001–0.84]
GSE66357	MCF10A cells	3'-end†	75-bp paired end‡	0.17 (0.004-1.8)
GSE113197	Human breast	Full length	100-bp paired end	1.5 (0.00001- 4.4)
PRJNA396019	Human breast	3'-end¶	75-bp & 100-bp paired end	0.88 (0.002-20.6)

*Read depth is reported as the median number of reads in millions per cell with the range in brackets.
†Sequencing was performed after a novel printing and RNA-capture approach[4].
‡Paired-end reads were collected asymmetrically, with ~20 bp barcode in read 1 and ~60 bp sequence in read 2.
¶Single-nucleus RNA-seq.

Supplementary Table S2. Primers used in this study.

Target gene	Forward	Reverse
Apoe1	GGTTCGAGCCAATAGTGGAA	GCCAGAGAGGTGCTTGAGAC
Chga	CTCGAGTAGGGCTGCTTCC	AGTTGTGCCCCATGGATAAG
ERCC4	ACTACTGGGGCGAGTATTCC	GGCAAGCCGGTGATTTTATCT
ERCC9	CTGTGTGTGATTCTCGAGCG	CTGTATATCGCGTTGCAGGG
ERCC19	ACTTGGAACGCTGCATAAGG	ACTTGGAACGCTGCATAAGG
ERCC28	GTGGGGAGTTAATCGAGCTG	ACTTGGAACGCTGCATAAGG
ERCC35	CTGAACGAGGGAGTGCTTGT	TGACCCATGGAGTGTTCTCA
ERCC42	AGCGTTTAAAGACAAAGCTCCA	ATCGACCCATTGAGAGAGCC
ERCC44	TTCGCCGGTTAGGTAAGGTA	ATCCGGTTATCCATGACGAG
ERCC60	GTGTGAAGTGGCATGTTTGG	CGATACAACCGTTCCAAGAAA
ERCC62	GCAAAAACGGCTATTGAAGC	TTTTGATGGAACTTTTTCTCCA
ERCC74	TGCTCATCCCTACTCATAACCC	CGCTTTGATATTCTCTGCATCCT
ERCC76	TGAGAGATCACGTGGACCAA	ATCTTGGCAGCTAACGATGC
ERCC85	ACTTGGAACGCTGCATAAGG	ACTTGGAACGCTGCATAAGG
ERCC92	TCCGTGTGTCGCGTAATACT	TCGATACGAGAGGGGATCAG
ERCC95	GAAACCCTACTTGCCTGCTG	CGATAGCGGTTAAGCCAGAG
ERCC108	TCAGAACGACTCGGTAGCTC	AGCGCACAGTCTCGTCATAT
ERCC111	CAATTGGGCCTCGTCTGAAC	TGACGTCATGGATCTCGCTT
ERCC113	ACCCGACTTTTGGGTTTAGG	TACCAACTAGGAAGAGCGCC
ERCC116	GATTATGGCACATGCACTCG	ACAGTCTCGACAGCAGCTCA
ERCC126	CTCATGTGCATGTCCAGAGC	CTCATGTGCATGTCCAGAGC
ERCC130	GCCTCTCTTGCTCAGTGTCC	TGGTCAGTGCGAAATTTGAG
ERCC131	CATGTGCTCATGTTGGGAAC	CATGTGGACAGGAGCCAATA
ERCC136	GCAATTGCGCTTGGTTCAAA	GTGTTAATACCCGCACGCTT
ERCC145	GGTTTTGTGTTAGCAGCGGA	GCCCGGTTCGATCCAAATTT
GAPDH	AACGTGTCAGTGGTGGACCT	TCGCTGTTGAAGTCAGAGGA
Gapdh	GGCATTGCTCTCAATGACAA	GCCTCTCTTGCTCAGTGTCC
PARN	GTTTGTTTGGATGGCGGTCT	TCTATCTGAAGCGGGTGGAG
Ppt1	GAGCATGGACTCCCTAGTGC	TGCCCTTAACCCTCATCCT

SUPPLEMENTARY REFERENCES

1 Wang, L. & Janes, K. A. Stochastic profiling of transcriptional regulatory heterogeneities in tissues, tumors and cultured cells. *Nat. Protoc.* **8**, 282-301, doi:10.1038/nprot.2012.158 (2013).

2 Peng, G. *et al.* Spatial Transcriptome for the Molecular Annotation of Lineage Fates and Cell Identity in Mid-gastrula Mouse Embryo. *Dev. Cell* **36**, 681-697, doi:10.1016/j.devcel.2016.02.020 (2016).

3 Pereira, M. *et al.* Direct Reprogramming of Resident NG2 Glia into Neurons with Properties of Fast-Spiking Parvalbumin-Containing Interneurons. *Stem Cell Reports* **9**, 742-751, doi:10.1016/j.stemcr.2017.07.023 (2017).

4 Bose, S. *et al.* Scalable microfluidics for single-cell RNA printing and sequencing. *Genome Biol.* **16**, 120, doi:10.1186/s13059-015-0684-3 (2015).

SUPPLEMENTARY NOTE: STEP-BY-STEP PROTOCOL

In situ 10-cell RNA sequencing in tissue and tumor biopsy samples

Shambhavi Singh[1†], Lixin Wang[1†], Dylan L. Schaff[1], Matthew D. Sutcliffe[1], Alex F. Koeppel[2], Jungeun Kim[3], Suna Onengut-Gumuscu[4], Kwon-Sik Park[3], Hui Zong[3], Kevin A. Janes[1,4,5]*
[1] Department of Biomedical Engineering
[2] Bioinformatics Core
[3] Department of Microbiology, Immunology & Cancer Biology
[4] Center for Public Health Genomics
[5] Department of Biochemistry & Molecular Genetics, University of Virginia, Charlottesville, VA. 22908

* To whom correspondence should be addressed. Tel: +1 434 243 2126; Fax: +1 434 982 3870; Email: kjanes@virginia.edu
† The authors wish it to be known that, in their opinion, the first two authors should be regarded as joint First Authors

These protocols are maintained and updated at http://bme.virginia.edu/janes/protocols/

Fresh cryo-embedding of tissue samples for laser-capture microdissection

1. Cut tissue sample of interest to the size of the appropriate cryomold (VWR #25608-922).
   - *(Optional for fluorescence-guided LCM) After tissue isolation, confirm fluorescence using a stereomicroscope equipped with a fluorescence lamp and collect images if desired*
2. Cover the base of small cryomolds (VWR #25608-922) with ~1 mm thickness of Neg-50 embedding medium (VWR #84000-154).
   - *Make sure that the Neg-50 embedding medium lies as flat as possible at the base of the cryomold; if a meniscus forms, this will create an air pocket in later steps that will cause the block to crack in half during sectioning*
3. After the embedding medium has settled uniformly at the base of the cryomold, place the tissue into the embedding medium with a pair of forceps.
   - *Try to avoid air pockets underneath the sample; if an air pocket forms, the block may crack during sectioning*
4. Fill the remainder of the cryomold with Neg-50 and snap freeze the cryomold in a dry ice-isopentane bath. Keep the embedded samples on dry ice and embed the remaining samples.
5. Wrap the embedded samples in tinfoil and store at –80ºC for 6–12 months or more. Isopentane can be stored at room temperature and reused indefinitely (it should not be disposed of down the sink).

Frozen sectioning of freshly cryo-embedded samples for laser-capture microdissection

1. Transport embedded samples on dry ice to the cryomicrotome.
2. Place sample in the cryomicrotome box and equilibrate the box temperature to –24ºC.
   - *Higher box temperatures (e.g., –20ºC) are acceptable but can cause distortion of the tissue during sectioning*
   - *Slide rack must be inside the cryomicrotome box to store slides after sectioning*
   - *Keep the slides at room temperature*
3. Replace the microtome blade (if disposable).
4. Wipe the microtome blade and anti-roll bar with a Kimwipe moistened with both ethanol and RNAse Away.
   - *RNAse Away by itself will freeze to the blade*
   - *Be careful not to cut yourself when cleaning the blade*
5. Pop the embedded sample out of the cryomold, mount on the sectioning platform with Neg-50, and freeze until the sample has solidified on the sectioning platform.
6. Mount the sectioning platform on the cryomicrotome and cut trim sections until the tissue is visible on the cutting surface.
   - *Tissue will appear off-white, brown, or light pink against the white Neg-50*
7. Cut 8 µm sections, adjusting the anti-roll bar to prevent the section from accordioning. Use a brush to start the section, lay down the anti-roll plate, and continue sectioning slowly and evenly through the tissue with the vacutome on sectioning mode at 100%.
   - *Do not graze sections (subsequent section will be 16 µm instead of 8 µm)*
   - *Alternatively, one can omit the anti-roll bar and use the brush technique*
   - *8 µm sections are roughly one-cell thick to allow for the maximum recovery of biological material after microdissection*
8. Wick sections onto Superfrost plus slides (VWR #48311-703) and move the slide immediately to the slide rack inside the cryomicrotome box.
   - *Place the slide inside the cryomicrotome box for ~10 seconds before wicking the section*
   - *The section should just barely wet onto the slide with intermittent warming from a finger (overwetting will cause fluorescence to diffuse away from the labeled cells)*
   - *Do not dry the slide at room temperature (this will ruin the RNA integrity in the tissue)*
   - *To wick a second section per slide, warm the back of the slide with your finger for a few seconds before wicking (do not warm the part of the slide that contains the existing section)*
9. Continue cutting and wicking sections until good cuts are no longer possible.

- *One embedded sample typically yields 30-60 slides with two sections per slide*
- *Do not return a half-sectioned sample to –80ºC (the exposed sample will dehydrate and become damaged). If necessary, cover exposed samples with Neg-50 before storing.*

10. Move the slide box containing the frozen sections to dry ice and store at –80ºC for up to one month.

Cryosection processing for laser-capture microdissection

For fluorescence-guided LCM:

1. Fix 8 µm frozen sections in 70% ethanol for 15 sec.
    - *Move slides directly from –80ºC to ethanol (do not allow slides to warm to room temperature)*
    - *Process no more than four slides simultaneously*
    - *Use slide forceps to transfer slides from jar to jar. Do not use a rubber slide holder (the holder transfers liquid from jar to jar, which prevents dehydration).*
2. Dehydrate in 95% ethanol for 15 sec, then in 100% ethanol for 1 min.
3. Remove ethanol with a xylene dip for 2 min.
    - *A second xylene clearing is generally not necessary and will increase the likelihood of collateral pickup during microdissection*
4. Air dry 5–10 min and store slides in a dessicator.
    - *Another set of four slides can be started at this point, if needed*
    - *Ethanol can be reused for multiple slide sets, but should be discarded at the end of staining*
    - *Tissue should appear white, indicating light adhesion to the slide*
5. (Optional) Verify tissue fluorescence with an inverted fluorescence microscope.
6. Proceed immediately to laser-capture microdissection

For brightfield LCM:

1. Fix 8 µm frozen sections in 75% ethanol for 30 sec.
    - *Move slides directly from –80ºC to ethanol (do not allow slides to warm to room temperature)*
    - *Process no more than four slides simultaneously*
    - *Use slide forceps to transfer slides from jar to jar. Do not use a rubber slide holder (the holder transfers liquid from jar to jar, which prevents dehydration).*
2. Transfer to distilled water for 15 sec.
3. Stain with a few drops of nuclear fast red (Vector Laboratories #H-3403) containing 1 U/ml RNAsin Plus (Promega #N2611) for 15 sec.
    - *100 µl nuclear fast red + 2.5 µl RNAsin Plus is sufficient for four slides containing two sections per slide*
4. Transfer to distilled water for 15 sec (dip slide, remove slide, dip slide again).
5. Repeat Step 4 with a second jar of distilled water.
6. Dehydrate in 70% ethanol for 30 sec, 95% ethanol for 30 sec, and finally 100% ethanol for 30 sec.
7. Remove ethanol with a xylene dip for 2 min.
    - *A second xylene clearing is generally not necessary and will increase the likelihood of collateral pickup during microdissection*
8. Air dry 5–10 min and store slide in a dessicator.
    - *Another set of four slides can be started at this point, if needed*
    - *Replace the distilled water from Steps 2, 4, and 5 after each set of slides (the ethanol can be reused for multiple slide sets, but should be discarded at the end of staining)*
9. Proceed immediately to laser-capture microdissection

Laser-capture microdissection

1. Transport slides in a dessicator to a microdissector.
2. Turn on the instrument, spray hands with RNAse Away.
3. Load Capsure HS LCM caps (Arcturus #LCM0214) onto the instrument.
4. Detach LCM cap, focus laser, and begin dissecting at power = 50 mW and duration = 20 (tenths of milliseconds).
    - *If the sample has been appropriately dehydrated, this laser power should allow good capture and resolution (1–2 cells per laser shot)*

- *If the polymer does not wet after many shots, increase the laser power in 5 mW increments*
- *Imaging settings for camera capture*
    - *Phase A (no phase contrast)*
    - *Illumination tab: intensity = 50, gain = 0, diffuser = in*
    - *(Optional) Fluorescence tab: microscope lamp intensity = 45, microscope lamp camera gain = 100, fluorescence intensity = 25.*

5. If there is extensive collateral pickup from adjacent nondissected cells, press the LCM cap lightly on the adhesive of a Post-It note.
    - *Try to use the least adhesive Post-It note available, and press the cap as lightly as possible to minimize unintended sample loss*
6. Load the LCM cap onto the ExtracSure adaptor (included in #LCM0214) and store the cap-adaptor upside down at room temperature.
7. Proceed immediately to small-sample cDNA amplification.

<u>Small-sample cDNA amplification from microdissected cells</u>

1. Add 4 μl of digestion buffer to the ExtracSure adaptor.
    - *To include control ERCC spike-in transcripts, use recipe for "digestion buffer with ERCC spike-in mix"*
    - *Make sure that the digestion buffer completely covers the dissecting area of the LCM cap (lightly tap the base of the LCM cap, if needed)*
    - *Do not add more digestion buffer if planning on amplifying multiple samples from the same LCM cap (dilute afterwards in digestion buffer + digestion stop buffer)*
2. Cover the ExtracSure adaptor with a 0.5 ml thin-walled PCR tube (Applied Biosystems #N8010611).
    - *Some other 0.5 ml PCR tubes will fit tightly with the ExtracSure adaptor, but not all tubes (check for a tight fit beforehand with one of the spare ExtracSure adaptors)*
3. Incubate both the LCM cap and the remaining digestion buffer at 42ºC for 1 hr.
    - *Can use a dry-air incubator or a heat block that holds 15 ml tubes*
    - *For a heat block, make sure to cover the top of the block to maintain temperature*
    - *The proteinase K in the digestion buffer self cleaves at 42ºC, so the incubation will keep the digestion buffer similar to what is in the LCM cap, if dilutions of the samples are required*
4. Spin PCR tubes for 2 min at 2500 rpm on a benchtop centrifuge at room temperature.
    - *Higher centrifuge speeds will cause the ExtracSure adaptor to fly off from the PCR tube*
    - *Prepare digestion stop buffer during Step 4*
5. Quickly add 1 μl of digestion stop buffer to the sample and mix by pipetting.
    - *Make sure that PMSF has not precipitated from the digestion stop buffer*
    - *After stopping all the LCM caps, add the remaining digestion buffer to the remaining digestion stop buffer for the blank sample and any sample dilutions*
6. Vortex and centrifuge briefly.
7. (Optional) Dilute sample with digestion buffer + digestion stop buffer (mixed at a 4:1 ratio immediately beforehand).
8. Transfer 4.5 μl of the sample to a 0.2 ml thin-walled PCR tube (Applied Biosystems # N8010612) and place tube on ice.
9. Prepare a blank tube for the amplification:
    a. Add 4 μl of digestion buffer + digestion stop buffer and 0.5 μl of nuclease-free water to a 0.2 ml thin-walled PCR tube.
    b. Heat-denature at 65ºC for 1 min.
    c. Allow to cool at room temperature for 90 sec.
    d. Spin for 2 min at 14,000 rpm on a benchtop centrifuge at 4ºC.
10. Add 0.5 μl of SuperScript III (Invitrogen #18080-044), vortex briefly, and incubate at 50ºC for 30 min.
    - *For all incubations, use a thermocycler for more-accurate temperature control*
    - *Mix SuperScript III in the sample by pipetting up and down the 0.5 μl as best as possible and then vortexing at medium speed*
    - *Do not use the DTT that comes with the SuperScript III (causes precipitation of the $CoCl_2$ in the subsequent tailing reaction)*

11. Heat-inactivate by incubating at 70ºC for 15 min.
12. Place samples on ice and spin for 2 min at 14,000 rpm on a benchtop centrifuge at 4ºC.
13. Add 1 μl of RNAse H–Mg^{2+} mix and incubate at 37ºC for 15 min.
    - *Mix the RNAse H–Mg^{2+} mix in the sample by pipetting up and down the 1 μl as best as possible*
    - *Do not use the reaction buffer that comes with the RNAse H*
14. Place samples on ice and spin for 2 min at 14,000 rpm on a benchtop centrifuge at 4ºC.
15. Add 3.5 μl of 2.6× tailing buffer containing 0.2 μl of 400 U/μl terminal transferase per sample and incubate at 37ºC for 15 min.
    - *Mix the 2.6× tailing buffer + terminal transferase in the sample by pipetting up and down the 3.5 μl as best as possible*
    - *Label tubes for Step 18*
16. Heat-inactivate by incubating at 65ºC for 10 min.
    - *Prepare ThermoPol PCR buffer with everything except AmpliTaq and AL1 primer*
17. Place samples on ice and spin for 2 min at 14,000 rpm on a benchtop centrifuge at 4ºC.
    - *While samples are spinning, add AmpliTaq and AL1 primer*
18. Add 90 μl of ThermoPol PCR buffer and split sample into three 33 μl aliquots.
    - *Mix the ThermoPol PCR buffer in the sample by pipetting up and down the 90 μl as best as possible*
    - *Add 33 μl to two new 0.2 ml thin-walled PCR tubes and use the remainder as the third aliquot*
19. Run 25 cycles of PCR on a thermocycler containing a heated lid:
    a. 4 cycles of 94ºC denaturation (1 min), 32ºC annealing (2 min), 72ºC extension (2 min, with 10 sec increase at each cycle)
    b. 21 cycles of 94ºC denaturation (1 min), 42ºC annealing (2 min), 72ºC extension (2 min 40 sec, with 10 sec increase at each cycle)
    c. Indefinite hold at 4ºC
20. Pool the three splits into the original 0.2 ml PCR tube, vortex and centrifuge briefly, and run 5 cycles of PCR on a thermocycler containing a heated lid:
    a. 5 cycles of 94ºC denaturation (1 min), 42ºC annealing (2 min), 72ºC extension (2 min)
    b. Indefinite hold at 4ºC
21. Samples can be frozen at –20ºC for months to years.

<u>cDNA re-amplification for RNA-sequencing</u>

Before starting:
- Have small-sample cDNA material as template for reamplification
- Have access to a 96-well plate or SPRIPlate ring magnet (Beckman #A32782 or equivalent)

<u>Identification of optimum cycle threshold for reamplification:</u>
1. Set up Master mix-1 on ice (19 µl needed per sample).
2. Add 1 μl of 5×-diluted amplified cDNA to each well of an optically clear PCR strip tube (Bio-Rad #TLS0801 and #TCS0803) or hard-shell PCR plate (BioRad #HSP9601 and #MSB1001) (one well for each sample).
3. Add 19 μl of the master mix to each well on ice and mix by pipetting.
    - *Be sure to use barrier tips when mixing to avoid amplicon contamination of the pipette*
4. Run 40 cycles of qPCR:
    a. 1 min at 94ºC
    b. 2 min at 42ºC
    c. 3 min at 72ºC (fluorescence read at the end of the extension)
5. The optimum cycle for reamplification is when the first sample hits the middle of its exponential phase.
    - *Going longer than the middle of the exponential phase will overamplify the sample and cause loss of quantitative accuracy*
    - *The Nextera XT kit requires only 1 ng of cDNA, meaning that most samples will have acceptable yield even if there is a great disparity in the apparent abundances of cDNA material for different samples*
    - *High-Fidelity gives the highest yield at the end of the reamplification (do not substitute AmpliTaq)*

Reamplification and purification of small-sample cDNA:

1. Set up Master mix-2 on ice (99 µl needed per sample).
2. Add 1 µl of amplified cDNA to each well of a PCR strip tube or PCR plate (one well for each sample).
   - *Any thin-walled strip tube or plate will suffice at this step*
3. Add 99 µl of the master mix to each well and mix by pipetting.
   - *Be sure to use barrier tips when mixing to avoid amplicon contamination of the pipette*
4. Run the optimum number of cycles of PCR as determined in Part I:
   a. 1 min at 94ºC
   b. 2 min at 42ºC
   c. 3 min at 72ºC
5. Purify and concentrate the PCR reaction with AMPure XP beads (Beckman #A63880):
   a. Equilibrate AMPure beads at room temperature for 30 min before starting.
   b. Add 70 µl AMPure beads per 100 µl reamplification (0.7× volume), mix by pipetting 10 times, and incubate at room temperature for 15 min.
      - *The lower volume of AMPure beads compared to the manufacturer's protocol enables selective purification of amplicon >300 bp in size and the removal of AL1-oligo(dT) concatemers*
   c. Magnetize the samples at room temperature for 5 min.
      - *Depending on the magnet, some of the beads may stick to the upper walls of the tube; if using strip tubes, it is possible to tilt the strips and guide the beads to the bottom of the well*
      - *For the SPRIPlate ring magnet, the beads are supposed to stick to the walls of the tube*
   d. On the magnet, aspirate the supernatant with a gel-loading pipette tip, leaving 5 µl in the well.
      - *Leaving residual volume is important for plate magnets that pull the beads to the bottom of the well*
   e. On the magnet, wash with 200 µl of freshly prepared 80% EtOH for 30 sec and aspirate with a gel-loading pipette tip.
      - *80% EtOH is defined here as 8 ml 100% EtOH + 2 ml H_2O (no voluming up)*
      - *Add the 80% EtOH gently to the beads to avoid disrupting them*
   f. Repeat Step 5e, making sure to remove all the residual EtOH.
   g. Air dry the wells for 10 min at room temperature.
      - *It is important to dry the beads thoroughly to improve recovery, as EtOH will reduce the solubility of the bead-bound amplicon*
      - *However, it is also important not to overdry the beads, as the amplicon will not elute off the bead surface; avoid a "cracked" appearance of dried beads on the tube walls*
   h. Resuspend the beads in 10 µl of elution buffer (10 mM Tris-HCl, pH 8.5) and incubate for 2 min at room temperature.
      - *When dissolving in this small of a volume, it is important to rinse all the beads to the bottom of the well for maximum recovery*
      - *Be sure to use barrier tips when mixing to avoid amplicon contamination of the pipette*
   i. Magnetize the samples at room temperature for 1 min.
   j. Transfer the supernatant to a fresh PCR strip tube or PCR plate.
6. Repurify the PCR reaction with AMPure XP beads (Beckman #A63880):
   - *This second round of purification improves the amplicon purity from 85% to >95%*
   a. Equilibrate AMPure beads at room temperature for 30 min before starting.
   b. Add 7 µl AMPure beads per 10 µl purified sample (0.7× volume), mix by pipetting 10 times, and incubate at room temperature for 15 min.
      - *The lower volume of AMPure beads compared to the manufacturer's protocol enables selective purification of amplicon >300 bp in size and the removal of AL1-oligo(dT) concatemers*
   c. Magnetize the samples at room temperature for 5 min.
      - *Depending on the magnet, some of the beads may stick to the upper walls of the tube; if using strip tubes, it is possible to tilt the strips and guide the beads to the bottom of the well*
      - *For the SPRIPlate ring magnet, the beads are supposed to stick to the walls of the tube*
   d. On the magnet, aspirate the supernatant with a gel-loading pipette tip, leaving 5 µl in the well.
      - *Leaving residual volume is important for plate magnets that pull the beads to the bottom of the well*

e. On the magnet, wash with 200 μl of freshly prepared 80% EtOH for 30 sec and aspirate with a gel-loading pipette tip.
   - *80% EtOH is defined here as 8 ml 100% EtOH + 2 ml H_2O (no voluming up)*
   - *Add the 80% EtOH gently to the beads to avoid disrupting them*

f. Repeat Step 6e, making sure to remove all the residual EtOH.

g. Air dry the wells for 10 min at room temperature.
   - *It is important to dry the beads thoroughly to improve recovery, as EtOH will reduce the solubility of the bead-bound amplicon*
   - *However, it is also important not to overdry the beads, as the amplicon will not elute off the bead surface; avoid a "cracked" appearance of dried beads on the tube walls*

h. Resuspend the beads in 10 μl of elution buffer (10 mM Tris-HCl, pH 8.5) and incubate for 2 min at room temperature
   - *When dissolving in this small of a volume, it is important to rinse all the beads to the bottom of the well for maximum recovery*
   - *Be sure to use barrier tips when mixing to avoid amplicon contamination of the pipette*

i. Magnetize the samples at room temperature for 1 min

j. Transfer the supernatant to a fresh PCR strip tube or PCR plate

7. Use 1 μl of the 2x purified amplicon for quantification by Qubit assay, and store the remainder at –20ºC before Nextera XT tagmentation

<u>Amplicon quantification by Qubit assay (Life Technologies #Q32850) on the CFX96 instrument</u>

1. Prepare a seven-point standard curve by serially diluting the Qubit dsDNA BR Standard #2 (at 100 ng/μl) twofold in Qubit dsDNA Standard #1 (at 0 ng/μl) to yield 100, 50, 25, 12.5, 6.25, 3.1, and 1.6 ng/μl.
   - *Only 1 μl of each serial dilution will be used in the assay*
   - *Also be sure to include a blank of 100% Qubit dsDNA BR Standard #1*
2. Add 1 ul of each serial dilution or 2x purified amplicon to the base of an optically clear PCR strip tube (Bio-Rad #TLS0801 and #TCS0803) or hard-shell PCR plate (BioRad #HSP9601 and #MSB1001) (one well for each sample).
3. Prepare Qubit Working Solution by diluting Qubit dsDNA BR Reagent 200-fold in Qubit dsDNA BR Buffer.
   - *199 μl of Buffer + 1 μl of Reagent is enough for 8–9 samples*
4. Add 19 μl of Qubit working solution to each well and mix by pipetting 10 times.
5. Seal the strip tube or plate and read once on the CFX96 instrument at 22ºC after a hold at 22ºC for 2 min.
   - *The Qubit detection reagent has an excitation maximum at 510 nm and an emission maximum at 527 nm, which is close enough to SYBR Green (Ex/Em = 497/520 nm) to use any qPCR detection instrument*
   - *Our saved protocol is named "QUBIT"*
6. Export the ZPCR file from the instrument and load into the CFX Manager Software.
7. Once the PCRD file had been created, deselect all empty wells on the plate.
   - *Having only the active wells selected makes data export easier*
8. Go to Settings > Baseline Setting > No Baseline Subtraction to turn off the default background subtraction settings.
   - *It is crucial to export the raw RFU values or else the data will be uninterpretable*
9. Go to Export > Export All Data Sheets > Excel 2003.
   - *The file ending in "End Point Results.xls" will contain the end RFUs from the instrument for the selected wells*
   - *Export > Custom Export will export only one file of RFUs if set up correctly, although all the wells of the 96-well plate will be exported*
10. Open the exported RFU data in Excel, perform linear regression on the standard curve, and back calculate the DNA concentrations of the 2x purified amplicon samples.
11. Dilute a fraction of the 2x purified amplicon samples to 0.2 ng/μl in H2O for tagmentation with the Nextera XT kit.

Buffer recipes

- **Digestion buffer**
  57 µl nuclease-free H_2O
  20 µl 5× MMLV RT buffer ("First-strand buffer") (included in Invitrogen #18080-044)
  2 µl 1× stock primer mix
  1 µl 20 mg/ml proteinase K (Sigma #P2308)
  80 µl total volume
  Prepare digestion buffer on ice and use immediately
  Prepare proteinase K solution in nuclease-free H_2O and store in 20 µl aliquots at –20°C
  Do not add $CaCl_2$ to proteinase K solution
  After thawing proteinase K solution, keep at 4°C for up to one month
  Dilute 25× stock primer mix to 1× in nuclease-free H_2O before adding to digestion buffer

- **Digestion buffer with ERCC spike-in mix**
  17 µl nuclease-free H_2O
  40 µl 1:5*10^7 dilution of ERCC spike-in mix #1 (Ambion 4456740)
  20 µl 5× MMLV RT buffer ("First-strand buffer") (included in Invitrogen #18080-044)
  2 µl 1× stock primer mix
  1 µl 20 mg/ml proteinase K (Sigma #P2308)
  80 µl total volume
  (Same notes as above)

- **Digestion stop buffer**
  17 µl nuclease-free H_2O
  2 µl l SuperAse In (Invitrogen AM2696; previously used RNAse inhibitors discontinued)
  1 µl 100 mM PMSF (17.42 mg/ml) (Sigma #P7626)
  20 µl total volume
  Prepare digestion stop buffer at room temperature
  Add reagents in the order listed, mix very briefly, and use immediately (do not use if PMSF has visibly precipitated)
  Prepare PMSF solution in 100% EtOH shortly before use
  AEBSF cannot substitute for PMSF
  Add 1µl digestion stop buffer per 4 µl digestion buffer

- **25× stock primer mix** Store as 5 µl aliquots at –20°C
  15 µl nuclease-free H_2O
  5 µl 100 mM dATP (dNTP set: Roche #11277049001)
  5 µl 100 mM dCTP
  5 µl 100 mM dGTP
  5 µl 100 mM dTTP
  5 µl 80 OD/ml T_{24} (25 nmol synthesis from IDT)
  40 µl total volume
  Good for 6 months at –20°C
  Dilute 25× stock primer mix to 1× in nuclease-free H_2O before adding to cDNA-lysis buffer

- **RNAse H–Mg^{2+} mix**
  5 µl 5 U/ml RNAse H (Amersham #E70054Z)
  5 µl 25 mM $MgCl_2$
  10 µl total volume
  Prepare RNAseH–Mg^{2+} mix on ice and use immediately**2.6× tailing buffer** Store as 100 µl aliquots at –20°C
  363 µl nuclease-free H_2O

400 µl 5× Invitrogen terminal transferase buffer (Invitrogen #16314-015)
15 µl 100 mM dATP (dNTP set: Roche #11277049001)
778 µl total volume
Do not use the Roche 5× TdT reaction buffer that comes with the terminal transferase (lacks $CoCl_2$)
Add 0.2 µl of 400 U/µl terminal transferase (Roche #03333574001) per 3.5 µl 2.6× tailing buffer before adding to PCR tubes

- **ThermoPol PCR buffer:**
  71.35 µl nuclease-free H_2O
  10 µl 10× ThermoPol buffer (New England Biolabs #B9005S)
  2.5 µl 100 mM $MgSO_4$
  0.5 µl 20 mg/ml BSA (Roche #10711454001)
  1 µl 100 mM dATP (dNTP set: Roche #11277049001)
  1 µl 100 mM dCTP
  1 µl 100 mM dGTP
  1 µl 100 mM dTTP
  0.75 µl Taq polymerase (NEB #M0273L)
  0.75 µl Phusion polymerase (NEB #M0530L)
  0.15 µl 15 µg/µl AL1 primer (1 nmol synthesis from Invitrogen)
  90 µl total volume
  Prepare ThermoPol PCR buffer on ice in the order of reagents listed above
  Add polymerases (first) and AL1 primer (second) just before starting PCR

Reamplification for RNA sequencing

- **Master mix-1** Prepare fresh on ice
  2 µl 10× High-Fidelity PCR buffer without Mg^{2+}
  2.8 µl 25 mM $MgCl_2$
  0.04 µl 100 mM dATP
  0.04 µl 100 mM dCTP
  0.04 µl 100 mM dGTP
  0.04 µl 100 mM dTTP
  0.1 µl 20 mg/ml BSA
  0.06 µl 15 mg/ml AL1 primer
  0.2 µl High-Fidelity polymerase
  0.05 µl 100× SYBR green
  13.63 µl PCR-grade H_2O
  19 µl total volume

- **Master mix-2** Prepare fresh on ice
  10 µl 10× High-Fidelity PCR buffer without Mg^{2+}
  14 µl 25 mM $MgCl_2$
  0.2 µl 100 mM dATP
  0.2 µl 100 mM dCTP
  0.2 µl 100 mM dGTP
  0.2 µl 100 mM dTTP
  0.5 µl 20 mg/ml BSA
  0.3 µl 15 mg/ml AL1 primer
  1 µl High-Fidelity polymerase
  72.4 µl PCR-grade H_2O
  99 µl total volume
